# Supplementary material for: Molecular and topological reorganizations in mitochondrial architecture interplay during Bax-mediated steps of apoptosis
Source: eLife. 2019 Feb 4;8:e40712. doi: 10.7554/eLife.40712 (PMC6361589; doi:10.7554/eLife.40712)
Supplement: Transparent reporting form [file elife-40712-transrepform.docx]

***eLife’s* transparent reporting form**

We encourage authors to provide detailed information *within their submission* to facilitate the interpretation and replication of experiments. Authors can upload supporting documentation to indicate the use of appropriate reporting guidelines for health-related research (see [EQUATOR Network](http://www.equator-network.org/%20)), life science research (see the [BioSharing Information Resource](https://biosharing.org/)), or the [ARRIVE guidelines](http://www.plosbiology.org/article/info:doi/10.1371/journal.pbio.1000412) for reporting work involving animal research. Where applicable, authors should refer to any relevant reporting standards documents in this form.

If you have any questions, please consult our Journal Policies and/or contact us: [editorial@elifesciences.org](mailto:editorial@elifesciences.org).

**Sample-size estimation**

- You should state whether an appropriate sample size was computed when the study was being designed
- You should state the statistical method of sample size computation and any required assumptions
- If no explicit power analysis was used, you should describe how you decided what sample (replicate) size (number) to use

Please outline where this information can be found within the submission (e.g., sections or figure legends), or explain why this information doesn’t apply to your submission:

We did not pre-determine the sample size when the study was being designed.

For Bax recruitment by live-cell microscopy, the quantification is described in the Results section, “Bax clusters form regions of ribosome-exclusion in the cytosol”.

The sample sizes from which the electron tomography data were generated are listed in Supplementary Table T1.

All reported quantifications and sample sizes derived from the electron tomography data are described accordingly whenever they are mentioned in the Results, Figures or Figure legends.

How measurements and quantifications were performed is described in detail in the Material and Methods section “Quantification and statistical analysis”.

**Replicates**

- You should report how often each experiment was performed
- You should include a definition of biological versus technical replication
- The data obtained should be provided and sufficient information should be provided to indicate the number of independent biological and/or technical replicates
- If you encountered any outliers, you should describe how these were handled
- Criteria for exclusion/inclusion of data should be clearly stated
- High-throughput sequence data should be uploaded before submission, with a private link for reviewers provided (these are available from both GEO and ArrayExpress)

Please outline where this information can be found within the submission (e.g., sections or figure legends), or explain why this information doesn’t apply to your submission:

For live-cell confocal microscopy experiments, the number of repetitions is stated in the respective Materials and Methods section.

The number of experiments and sample sizes from which the electron tomography data were generated are described in the respective Materials and Methods section, and are listed in detail in Supplementary Table T1. Data and samples were excluded based on quality criteria described in the legend to Supplementary Table T1.

Electron tomograms of resin-embedded cells contain different mitochondria and different regions within cells, and thus represent biological replicates of GFP-Bax clusters and mitochondria. It is possible that occasionally two tomograms have been acquired on consecutive resin sections from the same cell region, and thus might contain different parts of the same GFP-Bax cluster or mitochondrion. Therefore, the number of electron tomograms does not strictly represent the number of biological replicates.

The same applies for GFP-Bax clusters in electron tomograms of vitreous sections.

Electron tomograms from different cryo-FIB milled lamellae are from different cells, and thus represent biological replicates of cells.

**Statistical reporting**

- Statistical analysis methods should be described and justified
- Raw data should be presented in figures whenever informative to do so (typically when N per group is less than 10)
- For each experiment, you should identify the statistical tests used, exact values of N, definitions of center, methods of multiple test correction, and dispersion and precision measures (e.g., mean, median, SD, SEM, confidence intervals; and, for the major substantive results, a measure of effect size (e.g., Pearson's r, Cohen's d)
- Report exact p-values wherever possible alongside the summary statistics and 95% confidence intervals. These should be reported for all key questions and not only when the p-value is less than 0.05.

Please outline where this information can be found within the submission (e.g., sections or figure legends), or explain why this information doesn’t apply to your submission:

For comparisons of mitochondria rupture size reported in Fig. 4M, matrix density reported in Fig. 5C, and distances to membrane for ATP synthase heads reported in Fig. 6J, the information can be found in the Results section, the respective Figure legend, and in the Materials and Methods section “Quantifications and Statistical Analysis”.

(For large datasets, or papers with a very large number of statistical tests, you may upload a single table file with tests, Ns, etc., with reference to sections in the manuscript.)

**Group allocation**

- Indicate how samples were allocated into experimental groups (in the case of clinical studies, please specify allocation to treatment method); if randomization was used, please also state if restricted randomization was applied
- Indicate if masking was used during group allocation, data collection and/or data analysis

Please outline where this information can be found within the submission (e.g., sections or figure legends), or explain why this information doesn’t apply to your submission:

Data were allocated to the different experimental groups based on the cell line, experimental condition or preparation and imaging method that they were sampling and this is stated each time the data are presented, both in the text and figures.

**Additional data files (“source data”)**

- We encourage you to upload relevant additional data files, such as numerical data that are represented as a graph in a figure, or as a summary table
- Where provided, these should be in the most useful format, and they can be uploaded as “Source data” files linked to a main figure or table
- Include model definition files including the full list of parameters used
- Include code used for data analysis (e.g., R, MatLab)
- Avoid stating that data files are “available upon request”

Please indicate the figures or tables for which source data files have been provided:

We include a single xls file named “Source Data” containing the numerical data presented in Figures 4M, 4N, 5C, 5J and 6J.
